# Characterization of dimensional, morphological and morphometric features of retrieved 3D-printed acetabular cups for hip arthroplasty

**DOI:** 10.1186/s13018-020-01665-y

**Published:** 2020-04-19

**Authors:** Lorenzo Dall’Ava, Harry Hothi, Johann Henckel, Anna Di Laura, Paul Shearing, Alister Hart

**Affiliations:** 1 Institute of Orthopaedics and Musculoskeletal Science, University College London, Brockley Hill, Stanmore, HA7 4LP UK; 2 Royal National Orthopaedic Hospital, Stanmore, HA7 4LP UK; 3 Electrochemical Innovation Lab, Department of Chemical Engineering, University College London, Torrington Place, London, WC1E 7JE UK

**Keywords:** 3D printing, Additive manufacturing, Orthopaedic implants, Hip arthroplasty, Porous acetabular cups

## Abstract

**Background:**

Three-dimensional (3D) printing of porous titanium implants is increasing in orthopaedics, promising enhanced bony fixation whilst maintaining design similarities with conventionally manufactured components. Our study is one of the first to non-destructively characterize 3D-printed implants, using conventionally manufactured components as a reference.

**Methods:**

We analysed 16 acetabular cups retrieved from patients, divided into two groups: ‘3D-printed’ (*n* = 6) and ‘conventional’ (*n* = 10). Coordinate-measuring machine (CMM), electron microscopy (SEM) and microcomputed tomography (micro-CT) were used to investigate the roundness of the internal cup surface, the morphology of the backside surface and the morphometric features of the porous structures of the cups, respectively. The amount of bony attachment was also evaluated.

**Results:**

CMM analysis showed a median roundness of 19.45 and 14.52 μm for 3D-printed and conventional cups, respectively (*p* = 0.1114). SEM images revealed partially molten particles on the struts of 3D-printed implants; these are a by-product of the manufacturing technique, unlike the beads shown by conventional cups. As expected, porosity, pore size, strut thickness and thickness of the porous structure were significantly higher for 3D-printed components (*p* = 0.0002), with median values of 72.3%, 915 μm, 498 μm and 1.287 mm (*p* = 0.0002). The median values of bony attachment were 84.9% and 69.3% for 3D-printed and conventional cups, respectively (*p* = 0.2635).

**Conclusion:**

3D-printed implants are designed to be significantly more porous than some conventional components, as shown in this study, whilst still exhibiting the same shape and size. We found differences in the surface morphologies of the groups, related to the different manufacturing methods; a key finding was the presence of partially molten particles on the 3D-printed cups.

## Background

Over 100,000 titanium acetabular and femoral components were implanted in patients in the UK in 2017 alone [1]. The majority of these implants were manufactured using conventional methods such as computer numerical controlled (CNC) machining of wrought bars. However, additive manufacturing (AM) technologies, also known as three-dimensional (3D) printing, are rapidly increasing in orthopaedics, particularly in producing off-the-shelf cementless porous acetabular components for total hip arthroplasty (THA) [2–5]. According to the United Kingdom (UK) National Joint Registry (NJR) [1, 6], approximately 11% and 13% of all uncemented cups for revision procedures performed in 2017 and 2018, respectively, were 3D-printed.

3D-printing enables the manufacture of complex porous structures and features that may provide enhanced fixation stability, compared to conventionally manufactured porous geometries [3]. Specifically designed pore shapes can be produced using 3D-printing, unlike traditional technologies where there is a limited control over the porous structure layout [7].The clinical rationale behind the use of 3D-printing for customized (patient-matched) implants was to overcome the limitations of conventional custom components, which could not address complex cases where the bone stock was very limited. Considering off-the-shelf cups, the main clinical rationale is to create porous structures that maximize bone integration, especially in revision procedures. Additionally, 3D-printing allows maximum control over the cup design, such as holes with reinforced edges and thinner cup walls for a given cup diameter, allowing surgeons to use larger femoral heads [8]

From an engineering perspective, 3D-printing limits the use of specialized tooling and complex assembly procedures, considering that both the dense and porous structures are built in one step. This reduces the cost of the final component, without affecting the increased complexity of the structure. If a more cost-effective streamline of production can be achieved with this technology, especially when a small number of parts is produced, however, the post-processing following the main manufacturing still represents a burden for the final cost [2, 7–10].

3D-printing enables the design and production of complex structures and optimized cup sizes, but its potential risks and clinical impact are still poorly understood [11].

Recent clinical studies of 3D-printed cups have shown good outcomes [12–15]; however, no laboratory studies have previously characterized the whole components. On the contrary, several studies have investigated the biocompatibility and suitability of 3D-printing to produce porous structure for orthopaedic applications, but these were limited to cylindrical or cubic specimens [7, 16–26],

The aim of this study was to better understand the properties of 3D-printed orthopaedic cups, characterizing their design and providing preliminary findings that could depict their interaction with bone. Using a set of conventionally manufactured cups as reference, we evaluated (1) a dimensional characteristic of the internal cup surface, which could impact the seating of the shell-liner; (2) surface morphology and porous structure on the backside of the cups, which are related to osseointegration; (3) the amount of bony attachment, as first indicator of the bone-implant interaction.

## Materials and methods

**Fig. 1:**
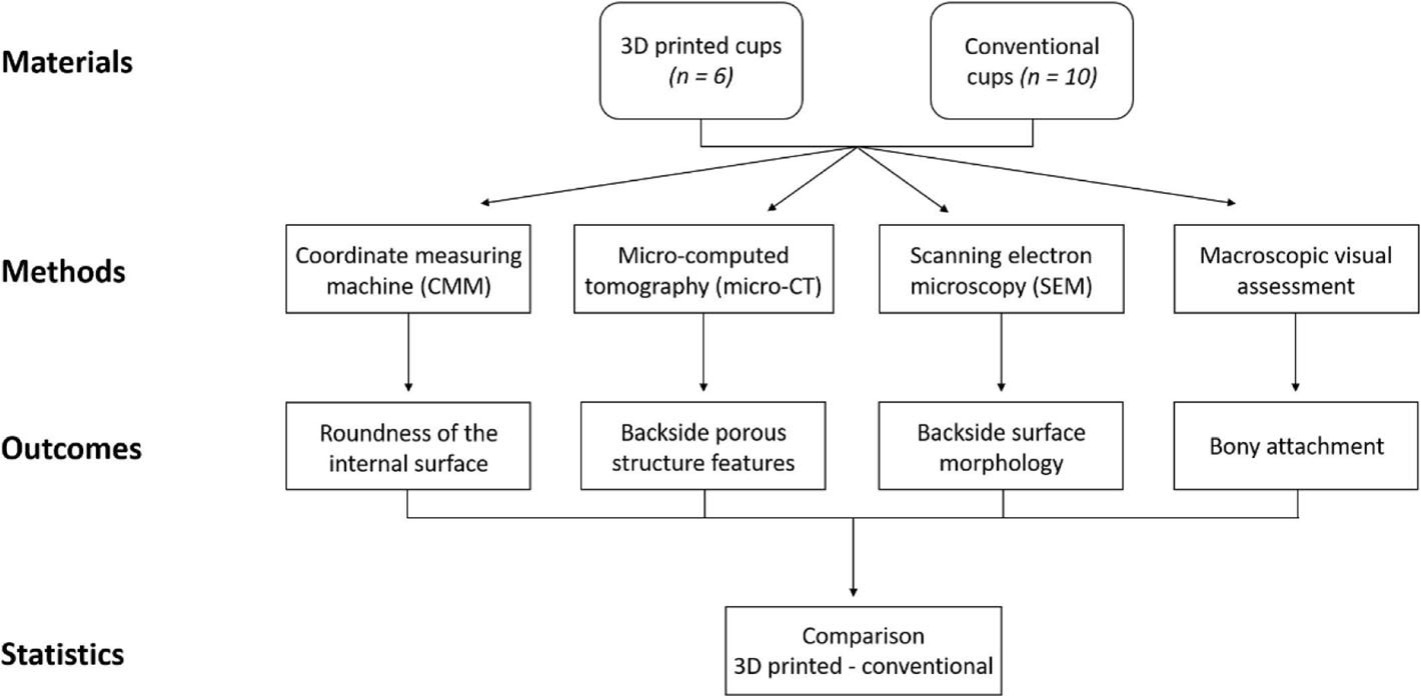
Flowchart of the study design

The study design is presented in Fig. 1. Institutional review board approval of the human protocol for this investigation was obtained (London-Riverside REC 07/Q0401/25).

### Materials

This study investigated 16 titanium-aluminium-vanadium (Ti6Al4V) acetabular cups received at our centre after being retrieved from patients. Whilst this was not a retrieval investigation, collection of retrieved implants enabled us to analyse designs from different manufactures. The cups were divided into two groups according to their manufacturing method: ‘3D-printed’ (*n* = 6; Delta TT; Lima Corporate, Udine, Italy) and ‘conventional’ (*n* = 10; Pinnacle Porocoat; DePuy Synthes, Raynham, USA). The 3D-printed implants had a median (interquartile range—IQR) size of 54 mm (50 to 56.5) and were produced using electron beam melting (EBM) [16]. This 3D-printed acetabular design is the most used in the UK according to the NJR [1]. The conventional cups had a median (IQR) size of 54 mm (52 to 56.5) and were manufactured forging and machining the acetabular shell and sintering the backside coating [27]; this uncemented conventional design is one of the most used in the UK [1].

Patients in the 3D-printed and conventional groups had median (IQR) ages of 61.1 (48.3 to 71.6) and 67.2 (63.3 to 70.1) years (*p* = 0.5941), a median (IQR) time to revision of 24.9 (21.1 to 46.8) and 49 (23.8 to 62.1) months (*p* = 0.296) and genders were 20% and 43% male, respectively. Reasons for revision included unexplained pain, aseptic loosening and infection for the 3D-printed group; adverse reaction to metal debris (ARMD), aseptic loosening and unexplained pain for the conventional one.

### Roundness of the internal cup surface

A Zeiss Contura (Carl Zeiss Ltd, Rugby, UK) coordinate-measuring machine (CMM) was used to measure the roundness of the internal cup surface. Measurements were taken using a 2 mm ruby stylus, recording 3 traces at different heights from the rim for each component, with an average of 14,756 points for each trace. The accuracy of the measurements technique was 2.2 + *L*/350 μm, where *L* is the measured dimension. Roundness values were automatically computed using the minimum zone circle method (difference between the radius of the most inside and outside points of the profile).

### Morphology of the backside surface

A scanning electron microscopy—SEM (Hitachi S3400-N, Tokyo, Japan) was used for detailed analysis of the surface morphology of the implants. Images were captured in secondary electron imaging (SEI) mode at 20 kV, with magnifications ranging from × 30 to × 180.

### Porous structure on the cup backside

Micro-computed tomography (micro-CT) analysis was used to investigate the morphometric features of porous structures on the backside of the implants. The method involved three steps: (1) micro-CT scanning of the acetabular components and data reconstruction, (2) segmentation and volumetric rendering of the micro-CT data and (3) measurement of morphometric parameters of the porous structures.

#### Micro-CT scanning

The implants were scanned using a micro-CT scanner (XTH 225, Nikon Metrology NV) set at 150 kV, with beam current of 70 μA. Scans included 3177 views in 0.11° of increment, with one frame per view and exposure of 1000 ms. Scanning of metallic object is affected by a beam hardening effect, which involves the attenuation of low energy photons (i.e., soft X-rays) whilst the X-rays beam moves through the absorbing material [28].. In order to reduce this effect, a physical Cu filter of 0.25 mm was used, located at the beam source; an algorithm (polynomial correction of fourth order) was also applied during the reconstruction process. Reconstruction of the scans was performed at full 35 μm isotropic resolution.

The choice of the micro-CT scanning parameters for Titanium samples lacks general conformity [28]; therefore, one of the implants was scanned three times using different sets of voltage and current: 80 kV, 200 μA; 100 kV, V100 μA; 150 kV, 70 μA. The values were chosen according to literature [29], spanning the range allowed by the scanner and avoiding saturation of the images. The results were compared semi-quantitatively, in terms of (1) presence of the peak corresponding to the metal object in the histogram plot of voxel count versus voxel intensity (grey level) and (2) appearance of the rendered volumes.

**Fig. 2:**
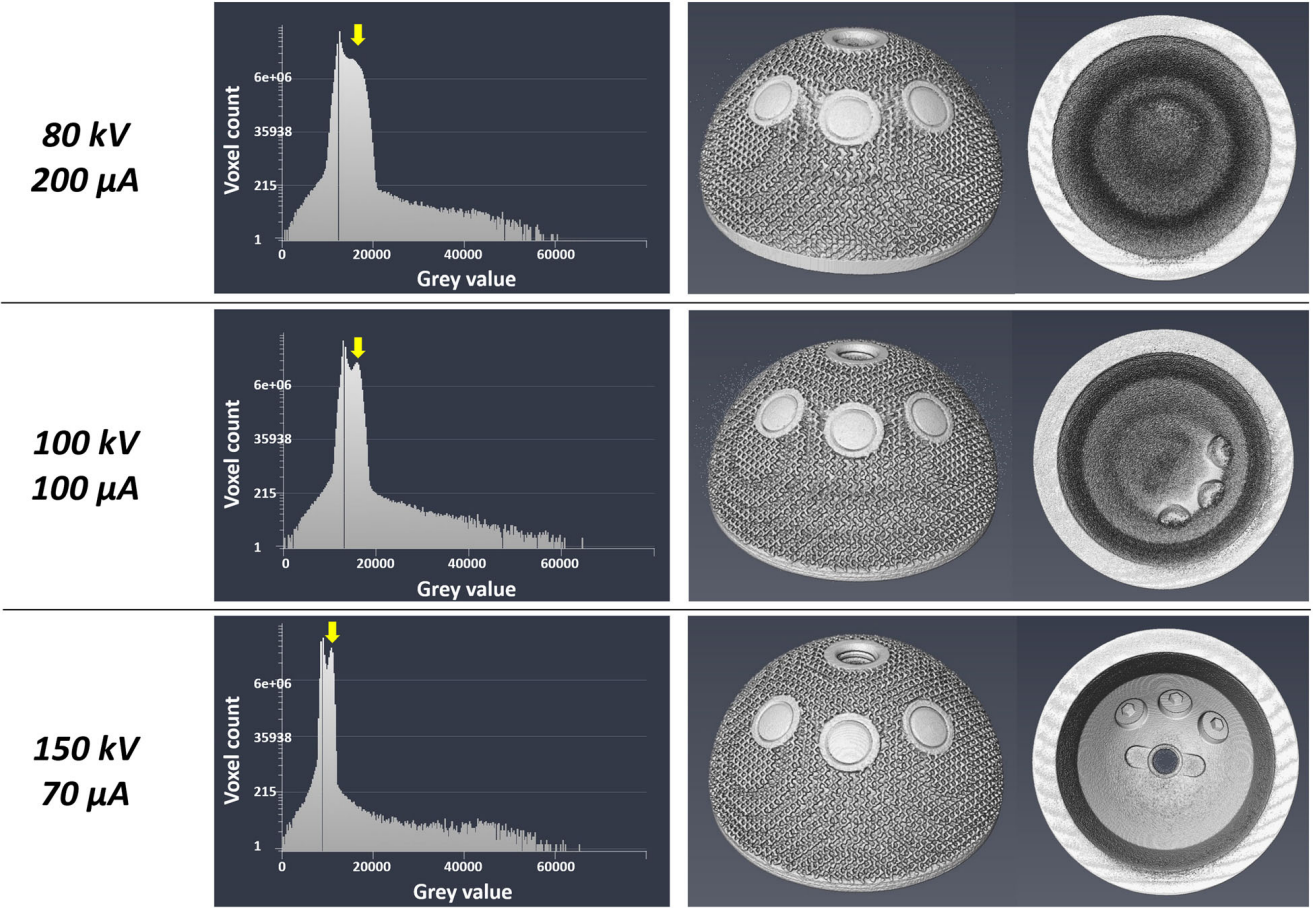
Images showing the outcomes of the optimization process of the micro-CT scanning parameters. From top to bottom, the investigated parameters were 80 kV, 200 μA, 100 kV, 100 μA and 150 kV, 70 μA. The set of values 150 kV, 70 μA was chosen because the peak corresponding to the metal (yellow arrow) in the voxel count histogram was higher compared to the same peak in the plots related to the other set of parameters. A higher peak suggested a better separation between voxels belonging to the background (air) and voxels belonging to the material. The reconstructed volume obtained with the values 150 kV, 70 μA provided a clearer representation of the implant compared to the others

The histogram plot showed a higher peak corresponding to the metal sample when using the set of parameters 150 kV, 70 μA (Fig 2), implying a better separation between voxels belonging to the background (air) and voxels related to the titanium sample. The rendered volumes confirmed the previous outcome, with all the features of the implant clearly visible without any noise. The set 150 kV, 70 μA was then chosen for scanning all the implants.

#### Segmentation and volume rendering

The reconstructed scans of the implants were segmented and rendered using a 3D micro-CT analysis software (Avizo 9.0, Thermofisher Scientific, US). The segmentation was performed by choosing a specific edge grey value for each implant, using an automatic segmentation process based on the ‘iso-50’ principle [28]. The threshold value corresponded to the mid-grey-level between the peaks that coincide with the irradiated materials (in our case titanium alloy and air as background) in the histogram plot of voxels count versus voxel intensity.

#### Measurement of morphometric parameters of the porous structure

The 3D reconstructed models were used to assess clinically relevant morphometric features of the porous structure of the implants: (1) *porosity*, which is the percentage of void space in a volume, (2) *pore size*, calculated as the diameter of the pores and (3) *strut thickness*, which is the dimension of the single parts composing the porous framework (in the conventional cups, these features were measured considering the approximate struts created by the packed beads that form the backside coating). Porosity and pore size represent the available space for bone ingrowth; strut thickness is the site for bone cell attachment. The *thickness of the porous structure* was also measured, representing the maximum penetration depth for bone tissue.

The measurements were performed using a 3D micro-CT analysis software (Avizo 9.0, Thermofisher Scientific, US) and a public domain software for image analysis (ImageJ 1.52a, Broken Symmetry Software) selecting volume of interest confining the porous network only and cross-sections of the implants. Overall, 85 measurements were taken on each component.

### Macroscopic analysis: visual assessment of tissue ongrowth

The area of bony tissue attachment was measured using a photogrammetric method including an imaging system (EOS 5D Mark II camera, Canon, Tokyo, Japan) and a public domain software for image analysis (ImageJ 1.52a, Broken Symmetry Software). Image calibration was performed by positioning a reference scale in the field of view. An overall percentage of bony attachment was calculated by averaging the measurements from four areas in which the cups were divided into; presence of bone was marked only when a white porous structure was visible (i.e. clear evidence of bony tissue).

### Statistical analysis

Statistical analysis was performed using Prism 7 (GraphPad, USA). The differences between 3D-printed and conventional cups in terms of roundness of the internal cup surface, morphometric features of the porous structures (porosity, pore size, strut thickness), thickness of the porous structure and bony tissue attachment were evaluated using non-parametric Mann-Whitney tests. The level of significance for all statistical analyses was *p* < 0.05. It should be noted that the small sample size in both groups could affect the statistical results, not allowing definitive conclusions to be drawn. Therefore, the outcomes from statistical analysis should only be considered as a descriptive indication of the comparison between 3D-printed and conventional cups.

## Results

The “Results” section is presented according to the location investigated on the acetabular components, starting with the analysis performed on the internal cup surface and continuing with the investigation of the backside of 3D-printed and conventional cups.

### Roundness of the internal cup surface

From the analysis of the internal cup surface using the CMM, a median (IQR) roundness of 19.45 μm (15.69 to 37.30) and 14.52 μm (12.59 to 16.79) were obtained for 3D-printed and conventional implants, respectively (*p* = 0.1114).

### Morphology of the backside surface

**Fig. 3:**
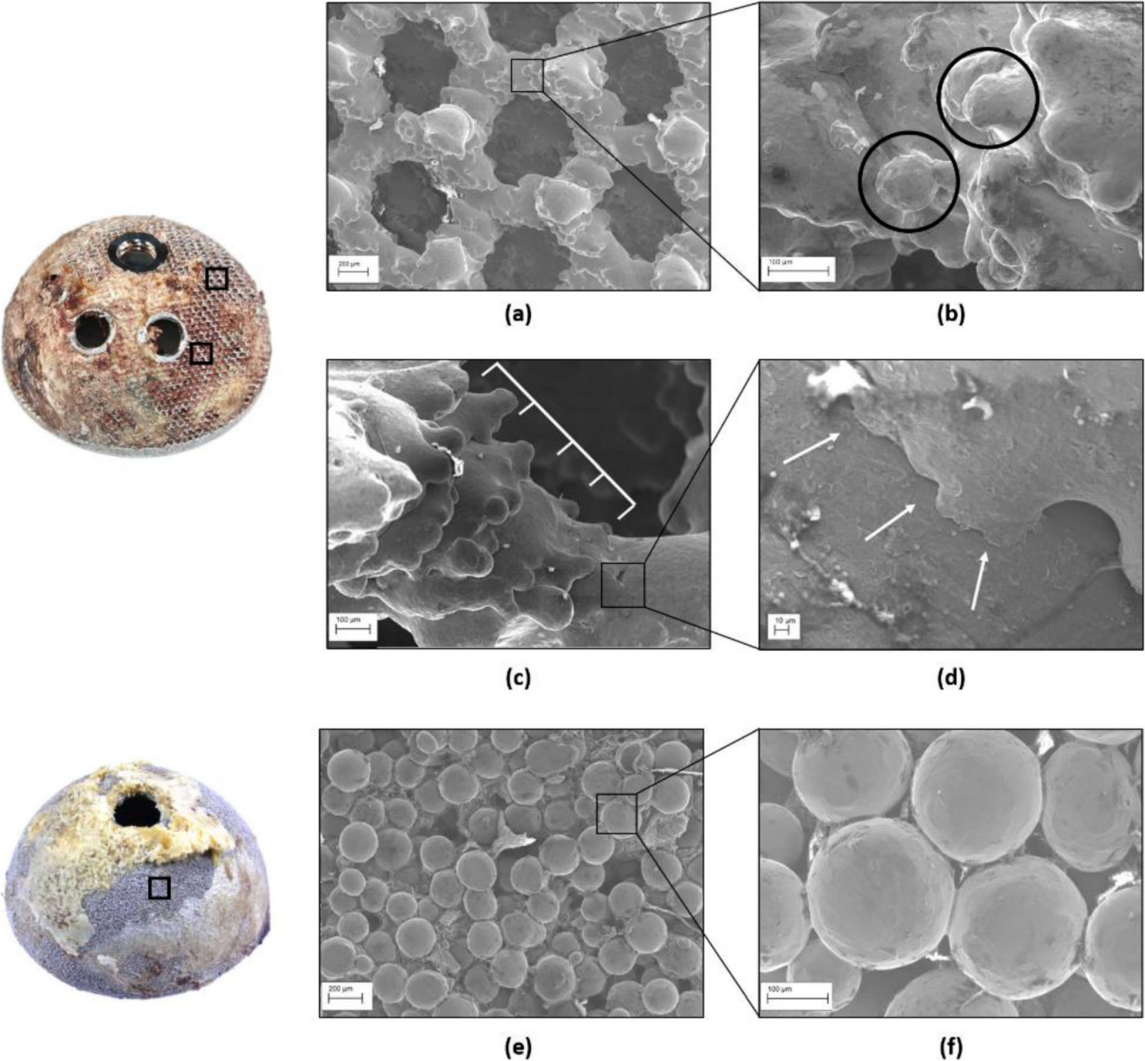
SEM images of a 3D-printed and a conventional implant. **a** The 3D-printed porous structure had a regular shape with clearly identifiable pores; **b** partially molten particles (black circles) were visible on the struts; **c** the ‘stair-step’ effect due to the layer-over-layer manufacturing and (**d**) the texture lines were also found at higher magnification. Differently, conventional cups showed **e** less clearly identifiable pores and **f** beads of uniform distribution along all the backside surface

As expected, the SEM analysis revealed differences in surface morphology between 3D-printed and conventional cups; the captured images are shown in Fig. 3.

At low magnification, the backside surface of the cups in the 3D-printed group exhibited a regular porous structure, with a repeated pattern of pores which approximately resemble hexagon-shaped unit cells. The implants in the conventional group showed a different morphology, with less visible pores between the packed beads that form the backside.

At higher magnification, all the 3D-printed acetabular cups showed particles of non-uniform location attached to the struts of the porous structure; the median size (IQR) of these particles was 73 μm (68 to 89).

Beads were also present on the conventional cups, but uniformly distributed across the backside surface and of bigger median size (IQR), equal to 208 μm (196 to 225).

The ‘stairstep’ effect due to the layer-over-layer manufacturing process and the texture lines between consecutive layers were also observed on the surface of the struts composing the porous structure of the 3D-printed cups; these features were not present on the surface of conventionally manufactured implants.

### Porous structure on the cup backside

**Fig. 4:**
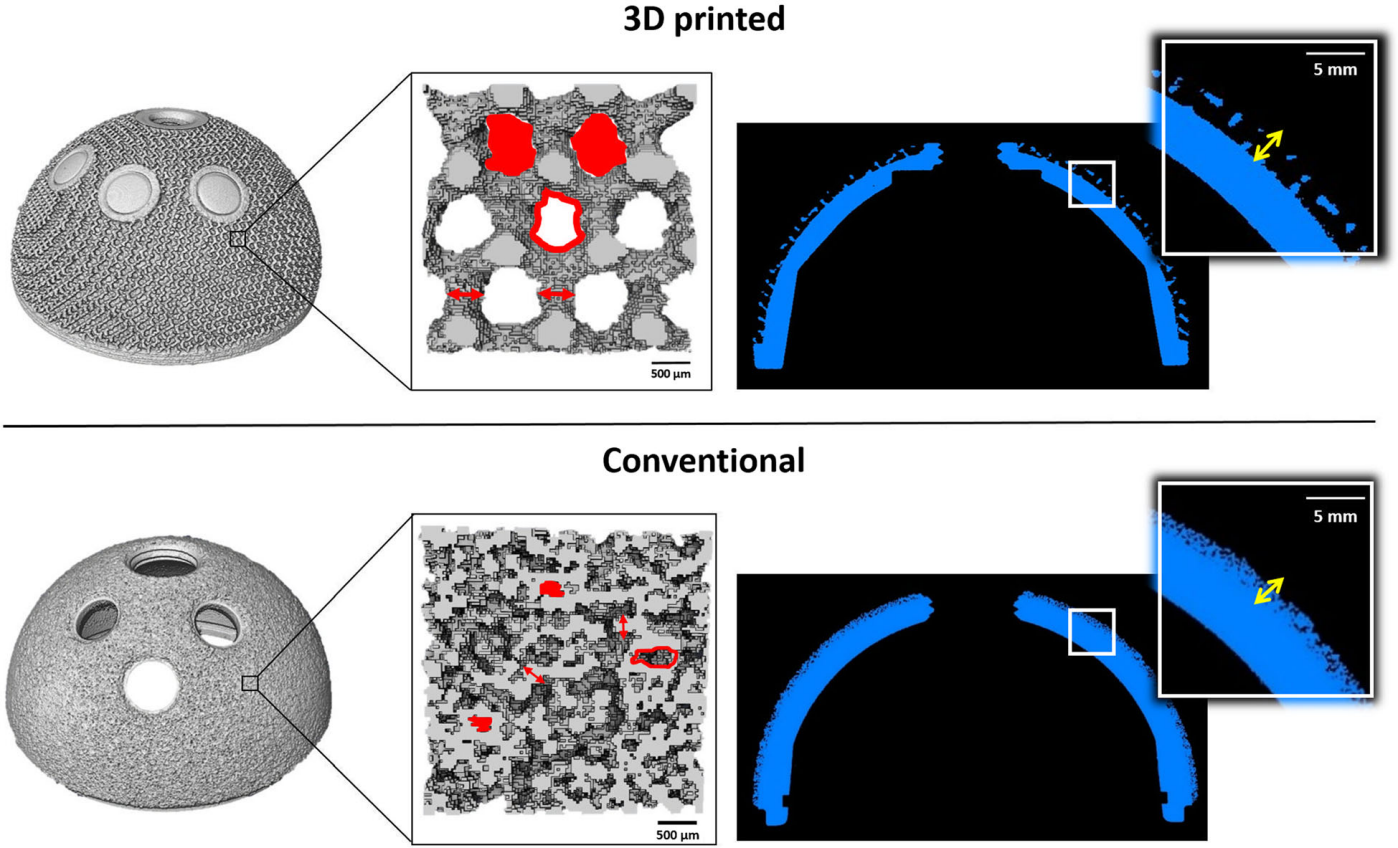
Images showing the outcomes from micro-CT analysis of 3D-printed (above solid line) and conventional (below solid line) cups. Left to right, the whole reconstructed implants and a zoom on the porous structures with the morphometric parameters depicted (porosity, pore size, strut thickness) are exhibited. Porosity is defined as the percentage of void volume (red filled shapes) over total volume; pore size is the diameter of the circle whose area equals the one of the red empty shapes; and strut thickness is indicated by the red arrows. A cross-section of the components and a zoom showing the thickness of the porous structure (yellow arrows) are also shown. 3D-printed cups showed a more porous and thicker porous backside structures

Micro-CT analysis confirmed that the acetabular components belonging to the two groups had very different porous structures, as observed from the SEM images. The 3D-printed cups showed a regular structure with recognizable pores, whilst the conventional implants exhibited an irregular backside surface, with identifiable circular beads and less visible pores (Fig. 4).

The median measurements of the morphometric features evaluated for the backside porous structures of the two groups of cups are summarized in Table 1.

**Table 1:** Median (IQR) measurements of the morphometric features of the porous structures of 3D-printed and conventional implants

Characteristic	3D-printed	Conventional	***p*** value
**Porosity, %**	72.3 (66.7 to 73.0)	32.7 (31.1 to 35.7)	0.0002
**Pore size, μm**	915 (854 to 954)	282 (265 to 293)	0.0002
**Strut thickness, μm**	498 (485 to 503)	240 (226 to 280)	0.0002

The porosity of 3D-printed cups was twice the value showed by conventional cups, and these differences were statistically significant (*p* = 0.0002). Similarly, the thickness of struts composing the porous framework of conventional cups was half the dimension exhibited by 3D-printed implants, with a significant difference between the two groups (*p* < 0.0002). Furthermore, the conventional components had pores which were three fold smaller in diameter than 3D-printed cups (*p* = 0.0002).

**Fig. 5:**
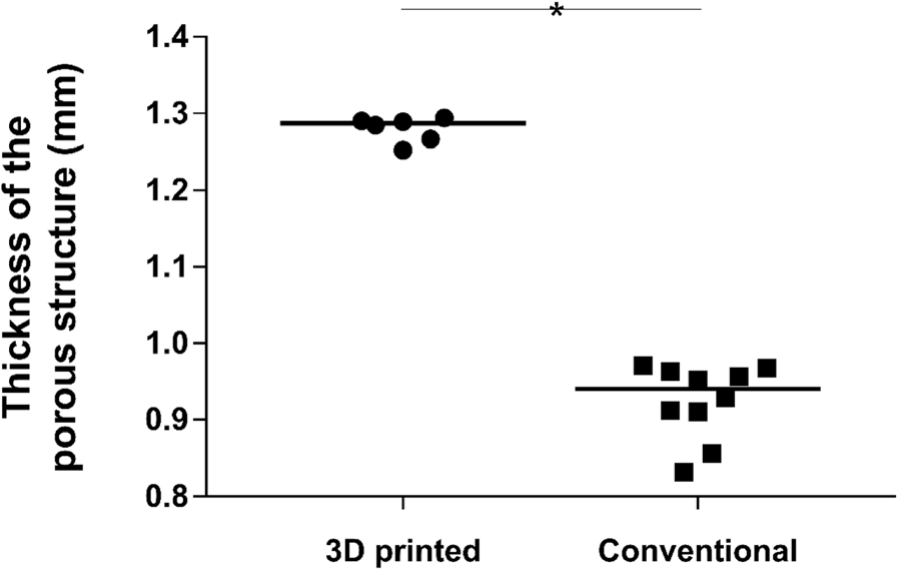
Dot plot showing the distribution of the values of thickness of the porous structure measured for the two groups. The solid line represents the median value. 3D-printed implants showed a significantly thicker structure than conventional cups (*p* = 0.0002)

3D-printed components also showed a thicker porous structure, with a median (IQR) value of 1.287 mm (1.263 to 1.291) compared to 0.940 mm (0.897 to 0.964) of the conventional cups. This difference was significant (*p* = 0.0002). The distribution of the thickness of the porous structure measured for both groups is presented in Fig. 5.

**Fig. 6:**
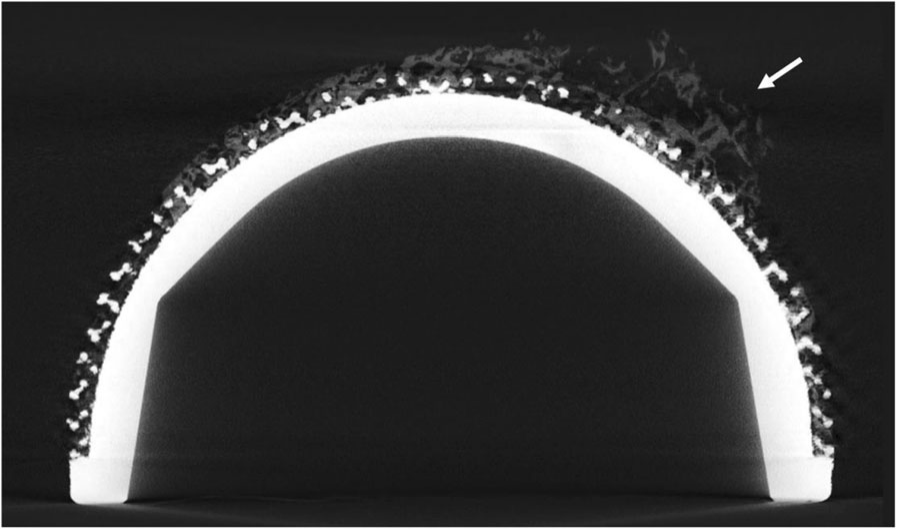
Image showing a CT cross-section slice of a 3D-printed cup with grey areas (white arrows) depicting presence of a material different from the Ti6Al4V alloy and background air

From the micro-CT slices, areas with grey-scale values different from the implant material (Ti6Al4V) and the background air were also identified (Fig. 6).

Comparison using Mann-Whitney test

### Macroscopic visual assessment of bony attachment

**Fig. 7:**
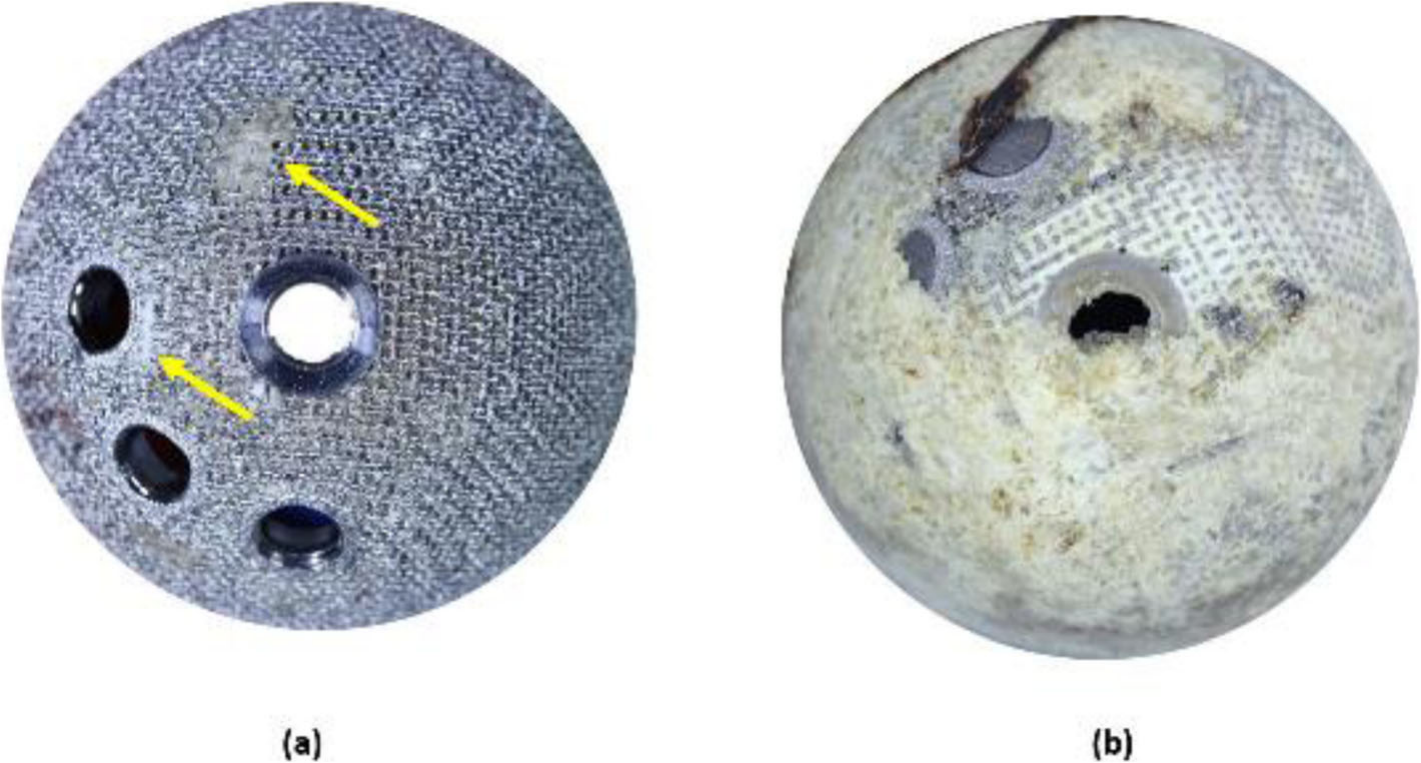
Image showing an example of **a** low and **b** high percentage of bony attachment on 3D-printed cups. The presence of bone in the image **a** is indicated by the yellow arrows

The 3D-printed and conventional cups had a median (IQR) bony attachment surface coverage of 84.9% (65 to 95.6) and 69.3% (65.1 to 93.9), respectively (*p* = 0.2635). Examples of high and low percentages of bony attachment are represented in Fig. 7.

Data related to each cup (gender, patient age, time to revision, morphometric parameters, thickness of the porous structure and bony attachment) are reported in Table 2 of the Appendix section.

## Discussion

This is one of the first studies to characterize 3D-printed orthopaedic acetabular components for THA; commonly used conventionally manufactured components were used as reference in order to highlight the features of these new types of implants. Conventionally manufactured cups with porous backside surfaces have been clinically adopted for roughly 40 years [30], showing good overall outcomes [31], thus being a valid benchmark to assess properties of 3D-printed implants that could influence their clinical outcomes. Although the clinical adoption of 3D-printed cups is not widespread yet, several 3D-printed acetabular designs are now available on the market and their use is expected to grow rapidly.

In this study, the use of retrieved cups allowed us to investigate designs that would be otherwise scarcely available, especially 3D-printed implants. A non-destructive method of analysis involving CMM, SEM and micro-CT imaging was used to investigate properties related to the seating of the shell-liner and features influencing osseointegration and implant stability.

As expected, we found differences in the morphometric properties of the porous structures, with twice the porosity, three times the pore size and twice the strut thickness in 3D printed cups. The porous structure was also thicker compared to conventional cups. Although these morphometric features are part of the actual 3D printing protocol, questions remain on what is optimal for bone-implants integration. Interestingly, we identified particles partially attached to the struts of the 3D-printed components; this is an unintended outcome of the 3D-printed process. The preliminary visual analysis of bony attachment revealed a similar percentage for the two groups of implants.

The comparable roundness of the internal surface of 3D-printed and conventional cups suggested that the different manufacturing methods did not influence this dimensional property. A wider range of values was however measured for the 3D-printed cups; it is unclear if this represents a true difference between the two groups, given the low number of implants included in this study. Overall, the internal surface of both 3D-printed and conventional cups is machined to obtain the specific tolerances prescribed by the manufacturer [27]. Moreover, comparable roundness between the two groups suggested that the cups did not deform in situ. The shell can deform upon insertion under certain conditions and an incorrect seating of the liner may cause its fracture (if ceramic) or adversely affect the fluid-film lubrication, which results in increased wear [32]. We found median values of less than 20 μm for both groups; although the dimensional tolerances for this feature were not available, however we could conclude that all cups showed a near-perfect round internal surface.

In terms of morphological properties, both groups exhibited the presence of particles. The key difference is that the beads present on the conventional implants are intended to form the porous structure (Porocoat) on the backside of the cups: titanium beads were sintered on the Ti6Al4V substrate (dense part of the shell) in a multilayered construct via high-temperature cycles in a vacuum furnace. The process induced solid-state diffusion with the substrate and between the beads [33]. Conversely, the partially molten particles on 3D-printed cups are a by-product of the manufacturing process: some of the powder particles that compose the starting feedstock of the 3D-printing process were not completely fused together. These particles, together with the texture lines, were previously observed in EBM-manufactured samples [34–36]. The texture lines were created by the layer-by-layer manufacture (‘stairstep effect’) and by small movement of the melt pool during the building process. The energy source (in this case electron beam) melted the metal powder, following the path provided by the computer-aided design (CAD) file and created areas of melted materials (pool) which are subjected to small movement [36].

In general, these morphological features provide a rough surface which may promote osteoblast cell adhesion and growth (osteoconduction), as previously demonstrated for this specific material construct [23, 24]. This falls under the material ‘contact guidance’ principle, whereby the cell movement and development is affected by the surface morphology of the implant [37]. However, a recent in vitro study by Xie et al. [38] suggested that the presence of the partially molten particles not only enhanced bacterial adhesion but also inhibited osteogenic activity of human bone mesenchymal stem cells, thus recommending the particles to be removed. The potential release of these particles cannot also be excluded, as suggested by Matouskova et al. [18]; this may lead to an increased risk of inflammatory reactions or decreased hemocompatibility [18], or to raised metal (titanium) levels in the blood. The high surface area of exposed metal given by the high porosity of the trabecular structure of 3D-printed components may increase this release process. To date, the only study that investigated the possibility of raised systemic metal (titanium) level in patient with Delta TT cups due to the highly porous architecture did not find any significant outcomes [39].

To the best of the authors knowledge, the issue of the partially molten particles has not been yet addressed by neither regulatory bodies, such as the Food and Drug Administration (FDA) in the USA, the European Medicines Agency (EMA) in the European Union or the Medicine and Healthcare Products Regulatory Agency (MHRA) in the UK, nor by standardization organizations, such as the American Society for Testing and Material (ASTM), the International Organization for Standardization (ISO) or the British Standards Institution (BSI). The FDA released general guidelines on additive-manufactured medical devices at the end of 2017 [40], and standards have been published on terminology, design, process, materials and test methods for 3D-printed parts [8]. However, an in-depth regulation is still missing.

As expected, differences were found between 3D-printed and conventional cups for all the morphometric parameters and the thickness of the porous structure. The Porocoat coating represents a traditional solution to obtain a porous backside on cementless acetabular cup, with titanium beads sintered to a Ti6Al4V shell. The morphometric properties of this coating, as defined by the manufacturer, include an average pore size of 250 μm, average volume porosity of 45% and depth of the coating of 0.762 ± 0.254 mm (*mean ± sd*) [27]; these are comparable to the values measured in our study. The influence of porosity and pore size on bone ingrowth and implant osseointegration is a controversial subject [41], but the morphometric values exhibited by the Porocoat coating fall into the range 100–400 μm that has been suggested by both in vitro and in vivo studies to be the optimum pore size [42–44]. In fact, the Pinnacle Porocoat acetabular component has been widely used in primary hip procedures performed in the UK during 2017 (28% of all uncemented cups) [1], and this coating has been applied to different acetabular cup designs for more than 30 years [27, 45], with good clinical outcomes [46].

The Delta TT acetabular cup has a highly porous backside that aims to achieve fixation in both primary and revision surgeries. The lattice structure of these cups is a regular matrix composed of multi-planar hexagons called Trabecular Titanium (TT), which have been previously characterized using cylindrical and cubic specimens. Marin et al. [16] considered two TT structure (‘small’ and ‘large’) in order to investigate the suitability of the AM process to produce porous structure for orthopaedic applications. They reported porosity of 63.2 ± 1.3% and 72 ± 0.8% and pore sizes of 0.64 ± 0.11 mm and 1.43 ± 0.07 mm for the former and the latter, respectively. These values are comparable to porosity and pore size measured in our study. Our measures of strut thickness were also found to be similar to another study by Regis et al. [26] that reported a value of 355 ± 10 μm. Suggested values of tolerance capability of the EBM technology (± 250/300 μm) support these statements [16, 26].

Clinical follow-up studies related to the Delta TT acetabular design showed good early and mid-term outcomes. Steno et al. [12] reported on the early results (mean follow-up period of 38.14 months) of 81 revision cases where only one re-revision was performed due to instability of the acetabular component. Perticarini et al. [13] found that 99.3% of the Delta TT cups used in 134 THA and 8 revision cases were radiographically stable at a mean follow-up period of 72.7 months; dislocation occurred in two cases, aseptic loosening in one patient. Munegato et al. [15] described good clinical and radiographic results at short- to mid-term follow-up (mean period 39.8 months), with three cases of dislocation.

In general, the aim of all cementless acetabular designs is to guarantee (1) good primary stability, which depends on the fitting between the cup and the host bone and on the coefficient of friction of the backside surface with bone [45] and (2) long-term fixation with bone. This is strongly influenced by morphometric features of the porous structure such as porosity and pore size. The human trabecular bone is made of a three-dimensional, interconnected, open-porous space, exhibiting high porosity (50–90%), big pore size (in the order of 1 mm) and trabecular (strut) thickness of hundreds of microns [47, 48]. The 3D-printed group showed morphometric values similar to those of trabecular bone. Interestingly, we identified a third material, different from Ti6Al4V and background air, in the cross-section slices of the 3D-printed cups obtained from the micro-CT investigation. These preliminary findings suggest that this may be the bone that grew into the porous structure of the cups, because only a material dense enough to attenuate the incoming X-rays would be visible in the CT slices. Further investigations will provide an insight into these observations.

Porosity, pore size and pore interconnectivity are key features that affect the interaction between the implant and bone tissue. These parameters are directly connected to the mechanical properties and the biological performance of the implant, influencing the recruitment, adhesion and proliferation of bone cells (*contact osteogenesis*), as well as the potential for vascularization and perfusion [41, 49]. The rationale for having porous structure on orthopaedic implants is to increase fixation and stability because of the mechanical interlocking created by bone growing into the structure [37]. The size of the pores determines which cells will colonize the material, directly affecting the progression of the osteogenic process. It has been reported that pores of 10–75 μm promote fibrous tissue growth; unmineralized bone tissue penetrates pores of 75–100 μm; and mineralized bone starts forming in pores of ~ 100 μm, with optimal bone infiltration in the range 100–500 μm [42–44, 50, 51]. A recent animal study by Tanzer et al. [25] described consistent bone ingrowth (> 50%) in a 3D-printed porous structure with 50 to 65% porosity and mean pore size of 450 μm. Therefore, it is accepted that lamellar bone formation requires a pore size of at least few hundred microns [49, 52]. However, other studies suggested that pore sizes in the range 500–900 μm facilitate bone ingrowth and can lead to higher infiltration or better cell response compared to structure with smaller pores [17, 19, 21]. It has also been shown that fibrous tissue formation occurs with pores bigger than 1000 μm [43, 48] and an upper limit in terms of pore size should be set also in relation to the mechanical properties. This is the reason why the optimal pore size for bone ingrowth is still a controversial subject in the literature [41]. Overall, optimal porosity and pore size alone do not entail successful implant osseointegration but constitute two of the main parameters within the ‘implant’ factors category. Other factors related to the ‘surgeon’ and ‘patient’ equally affect the final clinical outcome, such as surgical technique (e.g. anterior or posterior approach), implant choice and operation planning or patient age, clinical condition, bone quality and blood supply [49].

One of the main advantages of 3D-printing technology is the possibility to design and produce in a controlled way porous structure that resembles the properties of bone, as reported by several studies [2, 7, 16, 20, 26, 34, 35, 41]. Both regular (repeated unit cells) or irregular (stochastic) structures can be manufactured; topology optimization, where a mathematical model provides desired properties whilst satisfying prescribed constraints, can also be used [8, 41].

However, the potentially unlimited design freedom guaranteed by 3D printing does not imply that the quality of the final porous structure is suitable for medical application and this depends on the design itself and the fabrication parameters.

Despite the differences (i.e. higher porosity, pore size and thickness of the porous structure for 3D-printed cups), the acetabular designs showed comparable bony attachment, suggesting a similar behaviour with bone in situ. It is of note that 3D-printed cups exhibited a higher median value (85% vs 69%) despite the lower median time to revision (24 vs 49 months), although this difference was not significant. Further studies including more acetabular components would provide better insights on the topic.

The surgical technique applied to remove the cups was not considered to influence this outcome, as assessment of tissue within the porous structures at the surface level was also taken into account. In a retrospective comparative study, Swarts et al. [53] visually investigated the amount of tissue ongrowth on retrieved conventional acetabular designs, finding a strong association between type of shell porous surface and amount of tissue. Higher values were seen for cups with a backside coating made of beads and pore size around 250 μm, such as the Pinnacle Porocoat. Whilst there are clear differences in the porous structure of the two groups analysed in our study, it is of note that we saw evidence of good bone attachment on both designs.

To date, 3D-printed acetabular components have been mainly used in revision and re-revision surgeries where the patient’s bone quality is not as good as in a standard primary operation whose cause is osteoarthritis [1, 6]. This is probably due to the higher porosity and initial grip provided by the 3D-printed cups. It cannot be excluded that the number of primary operations adopting 3D-printed cups will increase, considering the decreasing age of patient undergoing primary hip surgeries, together with the raising age of the population, which leads to the need of longer-lasting implants.

A strength of this study was the use of micro-CT imaging on orthopaedic implants that are clinically used. Ho et al. [54] reported in 2006 the capabilities of this investigation method; since then, several studies [19–21, 35, 55] have used micro-CT to characterize the morphometric properties of 3D-printed Titanium porous structure (scaffold) for potential medical application, evaluating also both in vitro and in vivo biological outcomes. However, we are the first to combine the analysis of 3D printed implants for clinical application with micro-CT investigations. This method provided non-destructive, full three-dimensional information about shape, size and features of the as-produced components, overcoming obstacles experienced with retrieved samples like the presence of tissue attached on the surface. Other techniques, such as microscopy, can potentially provide similar data but limited to the outer surface of the structures, if the sample is preserved, or involving destructive preparation in order to obtain cross-sections to be analysed [16, 26].

A limitation of this study is the small number of implants. Only EBM-manufactured components were considered for the 3D-printed group, whilst another additive manufacturing technology (selective laser melting, SLM) is also used to produce this type of acetabular cups. The use of 3D-printed orthopaedic implants is not yet widespread, therefore limiting the number of samples available for analysis. All the implants analysed were retrieved from patients; the investigation of retrievals represents the only way to independently analyse components from different manufacturers having a fair number of samples under analysis.

## Conclusion

This was one of the first studies to characterize 3D-printed acetabular cups, using conventionally manufactured components as reference. We found differences in the porous structures, with higher porosity and pore size seen on 3D-printed cups. Morphological differences on the backside surfaces were also found, related to the different manufacturing methods; further investigations are needed in order to better understand the clinical impact of the partially molten particles on the surface of 3D-printed implants.

Future work involving larger and more heterogeneous cohorts of implants are needed in order to understand if the transition to 3D-printing techniques for mass production of orthopaedic implants can provide safe and well-performing outcomes.

## Data Availability

The datasets used and/or analysed during the current study are available from the corresponding author on reasonable request.

## Appendix

**Table 2:** Summary of the data related to the acetabular cups; morphometric parameters of the porous structure are reported as median (interquartile range) values

Case number	Gender	Age, years	Time to revision, months	Porosity, %	Pore size, μm	Strut thickness, μm	Porous thickness, mm	Bony attachment, %
**3D-printed**								
1	M	60.1	20.5	65.7 (64.8–67.5)	770 (756–787)	516 (455–560)	1.29 (1.17–1.4)	95.6
2	F	61.1	21.3	73.1 (71–74.1)	983 (962–999)	459 (424–513)	1.3 (1.18–1.41)	89.5
3	F	48.4	50.1	72.9 (72.1–74.1)	937 (918–953)	484 (470–518)	1.33 (1.19–1.4)	93.8
4	F	70.9	45.6	73 (71.3–74.1)	942 (934–958)	480 (459–542)	1.29 (1.23–1.36)	23.3
5	F	73.7	24.9	71.3 (70–74)	898 (877–915)	480 (467–523)	1.26 (1.13–1.36)	79
6	F	48.0	30.2	71.7 (71.3–73.2)	893 (818–907)	494 (459–512)	1.26 (1.22–1.33)	80.3
**Conventional**								
7	F	66	49	30.8 (28.1–33.6)	267 (245–335)	286 (235–327)	0.98 (0.91–1)	62.8
8	M	65	19	30.1 (27.9–37)	205 (192–239)	211 (182–245)	0.83 (0.77–0.88)	66.6
9	F	73.5	61.5	32.8 (29.3–35.1)	274 (234–329)	281 (254–308)	0.94 (0.85–1)	60
10	F	69.8	69	38.1 (34.1–39.6)	267 (226–307)	220 (197–279)	0.9 (0.85–0.96)	82.2
11	F	63	49	32.1 (30.1–35.9)	311 (226–384)	293 (264–306)	0.97 (0.88–1)	70.8
12	F	68.9	61	34.7 (33.3–38.5)	265 (220–353)	241 (224–269)	0.86 (0.79–0.9)	65.9
13	M	71	20	35.6 (32.2–38)	294 (242–321)	249 (215–268)	0.89 (0.84–0.98)	67.8
14	M	53	25	34.3 (28.6–36.9)	285 (252–359)	236 (223–290)	0.94 (0.9–1)	74.6
15	F	68.3	31	30.6 (29.3–37)	267 (205–299)	228 (204–248)	0.97 (0.89–1)	93.9
16	F	63.4	64	35.7 (32.4–39)	276 (220292)	220 (179–243)	0.96 (0.93–1)	93.4

## Abbreviations

3D: Three-dimensional
AM: Additive manufacturing
ARMD: Adverse reaction to metal debris
CAD: Computer-aided design
CMM: Coordinate-measuring machine
CNC: Computer numerical controlled
CT: Computed tomography
IQR: Interquartile range
NJR: National Joint Registry
SEM: Scanning electron microscopy
THA: total hip arthroplasty
Ti6Al4V: Titanium-6-aluminium-4-vanadium
TT: Trabecular titanium
